# Empowering Communities: Examining Korean Dementia‐friendly Community Policies through a Lens of Dementia Inclusive Societies Framework

**DOI:** 10.1002/alz.095429

**Published:** 2025-01-09

**Authors:** Soondool Chung

**Affiliations:** ^1^ EWHA WOMANS UNIVERSITY, Seodaemun‐gu, Seoul Korea, Republic of (South)

## Abstract

**Background:**

As the global prevalence of dementia continues to rise, South Korea faces the imperative of developing comprehensive policies to address the multifaceted challenges posed by this condition. Creating dementia‐friendly communities is important for the inclusion of patients with dementia (PWD) and their families. Therefore, the purpose of this study is to analyze dementia‐friendly community policies in Korea using the WHO’s dementia inclusive societies framework and to suggest ways to improve such policies.

**Methods:**

The WHO’s framework consists of key principles and actors such as key partners and target groups, etc.. The social environment, specifically Leading Institutions for Overcoming Dementia (LIOD) and Dementia Relief Members, was chosen as dementia‐friendly community policies in Korea.

**Results:**

The Dementia‐friendly Communities Initiative was started in 2017, aiming to support social activities of PWD. Firstly, the key principle of LIOD was to make communities safe for PWD and their families, with key partners being both for‐profit and non‐profit entities such as businesses, schools, universities, and libraries. They engage in activities, campaigns, educational programs, etc., related to dementia. The target groups were employees of business, students, and user of libraries. Secondly, the key principles of Dementia Relief Members were to promptly protect and report when encountering older individuals wandering the streets, and to encourage early dementia screening and increase awareness of dementia. Key partners were small business owners such as restaurant and convenience stores after their staff received Dementia Partners education. The target groups were owners and staffs of those shops.

**Conclusions:**

Dementia‐friendly community policy in Korea represents a critical step towards addressing the growing prevalence of dementia and promoting the rights and dignity of individuals living with the condition. However, there are some drawbacks to this policy. The system for sustaining the continuous activities of dementia LIOD is limited. Furthermore, the monitoring system for continuous activities of Dementia Relief Members is also inadequate. Dementia‐friendly establishments are only monitored for closure and changes in location, thus there is no effective management of continuous participation in activities. Further measures for improvement should be taken.

